# Electromyography-based fatigue assessment of an upper body exoskeleton during automotive assembly

**DOI:** 10.1017/wtc.2022.20

**Published:** 2022-09-19

**Authors:** Jason C. Gillette, Shekoofe Saadat, Terry Butler

**Affiliations:** 1 Department of Kinesiology, Iowa State University, Ames, IA, USA; 2 Lean Steps Consulting Inc., West Des Moines, IA, USA

**Keywords:** overhead work, shoulder, threshold limit values

## Abstract

The purpose of this study was to assess an upper body exoskeleton during automotive assembly processes that involve elevated arm postures. Sixteen team members at Toyota Motor Manufacturing Canada were fitted with a Levitate Airframe, and each team member performed between one and three processes with and without the exoskeleton. A total of 16 assembly processes were studied. Electromyography (EMG) data were collected on the anterior deltoid, biceps brachii, upper trapezius, and erector spinae. Team members also completed a usability survey. The exoskeleton significantly reduced anterior deltoid mean active EMG amplitude (*p* = .01, Δ = −3.2 %MVC, *d* = 0.56 medium effect) and fatigue risk value (*p* < .01, Δ = −5.1 %MVC, *d* = 0.62 medium effect) across the assembly processes, with no significant changes for the other muscles tested. A subset of nine assembly processes with a greater amount of time spent in arm elevations at or above 90° (30 vs. 24%) and at or above 135° (18 vs. 9%) appeared to benefit more from exoskeleton usage. For these processes, the exoskeleton significantly reduced anterior deltoid mean active EMG amplitude (*p* < .01, Δ = −5.1 %MVC, *d* = 0.95 large effect) and fatigue risk value (*p* < .01, Δ = −7.4 %MVC, *d* = 0.96 large effect). Team members responded positively about comfort and fatigue benefits, although there were concerns about the exoskeleton hindering certain job duties. The results support quantitative testing to match exoskeleton usage with specific job tasks and surveying team members for perceived benefits/drawbacks.

## 1. Introduction

There were 67,020 shoulder injuries in private industry that involved days away from work in the United States during 2019 (Bureau of Labor Statistics (BLS), 2020). The median days away from work was 27 for shoulder injuries, much higher than the 8 median days missed across all injuries (Bureau of Labor Statistics (BLS), 2020). Of these shoulder injuries, 65% were attributed to sprains, strains, tears, and tendonitis, and 63% were to overexertion and repetitive motion (Bureau of Labor Statistics (BLS), 2020). These data indicate that shoulder injuries are prevalent, result in missed work, and are consistent with work-related musculoskeletal disorders. For example, the shoulder accounted for 10% of total cases in automobile and light truck manufacturing, trailing only the hand and back for body part injured (Bureau of Labor Statistics (BLS), 2020). Specifically, shoulder flexion and abduction above 90° for 10% or more of the work cycle have been found to increase the risk of chronic shoulder disorders in automotive assembly (Punnett et al., 2000).

Upper body exoskeletons are a potential intervention to reduce shoulder fatigue in job tasks with elevated arm postures such as automotive assembly. There are mixed results depending upon the exoskeleton design and target application. One type of upper body exoskeleton design utilizes a mechanical arm or mechanical arms to assist the worker, particularly when holding objects such as tools or parts. For example, an exoskeletal vest with a mechanical arm reduced anterior deltoid activation and shoulder discomfort during bolt fastening (Rashedi et al., 2014), but increased low back perceived exertion during drilling (Alabdulkarim et al., 2019) and spinal loads during bolt fastening (Weston et al., 2018). An exoskeleton with two mechanical arms reduced anterior deltoid activation during lifting and stacking, but increased triceps brachii activation (Theurel et al., 2018).

Another type of upper body exoskeleton is anthropomorphic in design and assists the shoulder by directly supporting the arms. For example, an exoskeleton design that assists the shoulder through arm support reduced anterior deltoid and latissimus dorsi activation across static shoulder elevation angles from 30° to 150°, but increased trapezius activation at 150° (de Vries et al., 2019). Another arm support exoskeleton reduced anterior deltoid activation and NASA-TLX score during a pointing task with a 90° shoulder flexion angle (Maurice et al., 2020), and reduced anterior deltoid and descending trapezius activation during tightening and drilling tasks at 95° and 115° shoulder flexion (Schmalz et al., 2019). A third arm support exoskeleton reduced anterior deltoid activation during static and dynamic pointing and reaching tasks (Pacifico et al., 2020).

Arm support exoskeleton benefits and drawbacks may also depend on dynamic shoulder postures involved in the target application. For example, an arm support exoskeleton reduced anterior deltoid activation during drilling (Alabdulkarim and Nussbaum, 2019; Van Engelhoven et al., 2019). Another arm support exoskeleton reduced anterior deltoid activation, descending trapezius activation (Kim et al., 2018a), lumbar compression (Kim et al., 2018b), and shoulder/low back perceived exertion (Alabdulkarim et al., 2019) during drilling, but reduced shoulder range of motion (Kim et al., 2018b). This arm support exoskeleton also reduced anterior deltoid activation during wiring and reduced shoulder discomfort at overhead work heights (Kim and Nussbaum, 2019). Lab-based studies like these are beneficial for determining combinations of job tasks, arm postures, and tool weights that may benefit from exoskeleton usage.

On-site assessments of upper body exoskeletons provide “real world” data, although the types of measurements that are feasible on assembly lines may be limited. For example, an arm support exoskeleton reduced anterior deltoid activation and fatigue risk during agricultural equipment assembly (Gillette and Stephenson, 2019) and reduced anterior deltoid and trapezius activation during overhead automotive assembly (Iranzo et al., 2020). The combination of lab-based and on-site exoskeleton assessments has begun to define a range of job tasks where upper body exoskeletons may reduce muscular effort. While upper body exoskeletons may help to reduce the risk of work-related musculoskeletal disorders, more research is needed to document the benefits and risks of exoskeleton usage (Howard et al., 2019; McFarland and Fischer, 2019). Efforts to develop a roadmap for exoskeleton adoption (Crea et al., 2021) and a recent systematic review (De Bock et al., 2022) stress the importance of assessing exoskeletons in realistic field environments with skilled workers.

Fatigue risk values (Gillette and Stephenson, 2019) were calculated in this study by comparing electromyography (EMG) amplitudes to threshold limit values (TLVs) for localized muscle fatigue (American Conference of Governmental Industrial Hygienists (ACGIH), 2016). The ACGIH TLV was utilized to provide a single value that combines the intensity of muscle activity with how often the muscle is active to predict muscle fatigue. When used during field studies, the ACGIH TLV estimates if a job task is fatiguing without an exoskeleton and whether the job task remains above the TLV or drops below the TLV with exoskeleton usage. A previous study using this methodology assessed agricultural equipment manufacturing tasks that were less repetitive (cab assembly, hydraulic assembly) or involved heavier objects (parts handing, welding) than automotive assembly (Gillette and Stephenson, 2019). The current study was performed to assess if exoskeleton usage would be effective for reducing muscle fatigue in an occupational setting that is much more repetitive.

The purpose of this study was to determine if an upper body exoskeleton reduced muscle fatigue risk during automotive assembly job tasks at Toyota. Early results of this study have been previously reported (Gillette and Stephenson, 2018). This manuscript expands on that previous work by adding six additional team members, five additional assembly processes, and a revised ACGIH TLV analysis that includes the entire duty cycle curve (instead of just 10 and 50%). A secondary goal was to identify if there were job tasks that appeared to benefit more than others from exoskeleton usage and explore possible explanations for differences. Our hypothesis was that anterior deltoid activation and fatigue risk would be significantly reduced when using the upper body exoskeleton during automotive assembly tasks.

## 2. Methods

### 2.1. Participants

**Figure 1.**
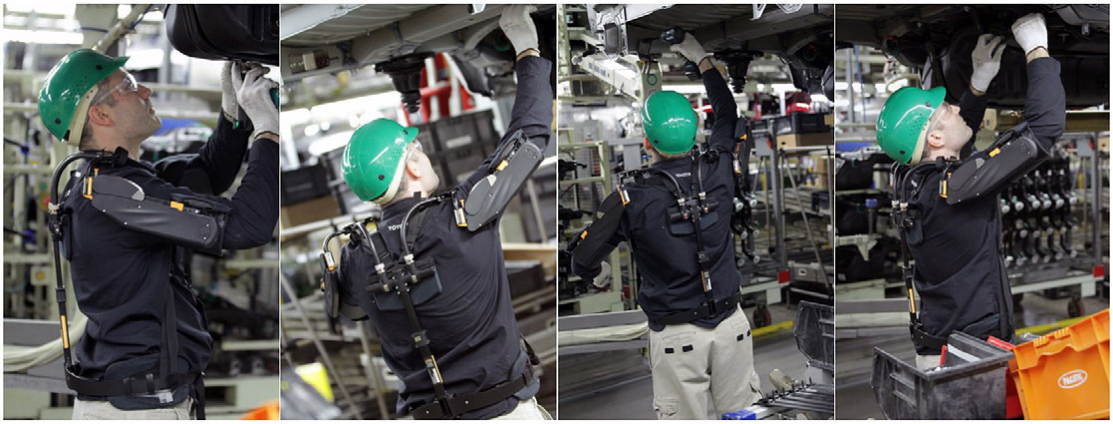
Representative postures for assembly processes #1–#4. See Table 1 for process descriptions.

Ergonomists and safety specialists at Toyota Motor Manufacturing Canada in Cambridge, Ontario identified 16 overhead assembly processes on two automotive assembly lines that might benefit from upper body exoskeleton usage. These assembly processes were selected because they involved repetitive elevated arm postures, and we were provided the Toyota Ergonomic Burden Assessment (TEBA) sheets for each process for secondary analysis. The TEBA sheets included how many seconds team members were expected to be in 60°, 90°, 135°, and 180° shoulder elevations for each process. Sixteen experienced male team members who worked on these assembly lines volunteered for this study. Participant mean (standard deviation) characteristics were age 35 (6) year, mass 87 (16) kg, and height 1.79 (0.05) m. To be included in the study, team members had to be trained and regularly perform the selected process and there had to be a replacement team member who could perform the process while the participant was being prepped for data collection and completing surveys. Ten team members were tested on two processes, five team members were tested on one process, and one team member was tested on three processes. Twelve processes were performed by two team members, and four processes were performed by one team member. Representative postures for assembly processes #1–#4 appear in Figure 1, and a description of the processes with the team members who performed each process appears in Table 1.

**Table 1.** Automotive assembly process descriptions and team members who performed each process

	Process description
1	Team members positioned fuel tank, tightened band bolts, performed visual checks, installed fuel inlet tube and filter pipe; and connected fuel lines. Elevated arm postures: 33%. Most common posture: 90° shoulder flexion, 90° elbow flexion. Cycle time: 115 s, repetitions: 6, duration: 11 min, 30 s. Team members #12 and #13 (both cassette #3)
2	Team members placed and installed various floor grommets; installed fenders and rocker panels; adhered stickers, installed wiring block, and adhered seal plate. Elevated arm postures: 47%. Most common posture: 135° shoulder flexion. Cycle time: 115 s, repetitions: 6, duration: 11 min, 30 s. Team members #12 and #13 (both cassette #3)
3	Team members installed radiator saddle, cross member, tow hook, insulator, and ball joints; and tightened engine bolts. Elevated arm postures: 31%. Most common posture: 60° shoulder flexion, 90° elbow flexion. Cycle time: 66.5 s, repetitions: 10, duration: 11 min, 5 s. Team members #6 (cassette #2) and #11 (cassette #3)
4	Team members installed fuel tank charcoal canister, connected outlet hose, wire harness, supports, and spacers; and performed visual checks. Elevated arm postures: 73%. Most common posture: 90° shoulder flexion, 90° elbow flexion. Cycle time: 115 s, repetitions: 6, duration: 11 min, 30 s. Team members #14 and #15 (both cassette #3)
5	Team members positioned fuel tank, installed fuel tank holding straps, fill tube, vent hose, and filter. Elevated arm postures: 27%. Most common posture: 135° shoulder flexion. Cycle time: 66.5 s, repetitions: 10, duration: 11 min, 5 s. Team members #1 and #7 (both cassette #3)
6	Team members installed rear left-hand absorber, tightened bolts, installed transmission cable and hole plugs; installed breather tube, and secured fuel lines. Elevated arm postures: 32%. Most common posture: 135° shoulder flexion. Cycle time: 66.5 s, repetitions: 10, duration: 11 min, 5 s. Team members #2 and #8 (both cassette #3)
7	Team members installed brake lines by removing them from a rack, raising them into position, clipping them in place, securing them, and connecting them. Elevated arm postures: 26%. Most common posture: 135° shoulder flexion. Cycle time: 66.5 s, repetitions: 10, duration: 11 min, 5 s. Team members #1 and #7 (both cassette #3)
8	Team members installed drive shaft and radiator hose; clamped and tightened clutch tube nuts; and installed and tightened center member to the cross frame. Elevated arm postures: 26%. Most common posture: 135° shoulder flexion. Cycle time: 66.5 s, repetitions: 10, duration: 11 min, 5 s. Team members #6 (cassette #2) and #11 (cassette #3)
9	The team members installed clutch cable, front subframe, shifter, and heat shield to the body. Elevated arm postures: 36%. Most common posture: 90° shoulder flexion, 90° elbow flexion. Cycle time: 66.5 s, repetitions: 10, duration: 11 min, 5 s. Team members #4 (cassette #2) and #5 (cassette #3)
10	Team members installed transport hooks and high voltage coil brackets; applied under coating, riveted heat insulators, and installed grommets. Elevated arm postures: 45%. Most common posture: 90° shoulder flexion, 90° elbow flexion. Cycle time: 115 s, repetitions: 6, duration: 11 min, 30 s. Team members #14 and #15 (both cassette #3)
11	Team member installed gas canisters, noise filters, washer hoses, antenna bracket, wiring, grommets, and ground wire. Part of work was seated. Elevated arm postures: 44%. Most common posture: 90° shoulder flexion, 90° elbow flexion. Cycle time: 115 s, repetitions: 6, duration: 11 min, 30 s. Team member #16 (cassette #3)
12	Team member positioned, secured, and installed sunroof or panoramic housing. Elevated arm postures: 32%. Most common posture: 60° shoulder flexion, 90° elbow flexion. Cycle time: 66.5 s, repetitions: 10, duration: 11 min, 5 s. Team member: #16 (cassette #3)
13	Team members installed air conditioning grommet, hose, shifter cable bracket, heat shields, and main fuel line; and tightened parking brake cable. Elevated arm postures: 30%. Most common posture: 90° shoulder flexion, 90° elbow flexion. Cycle time: 66.5 s, repetitions: 10, duration: 11 min, 5 s. Team members #2 and #10 (both cassette #3)
14	Team members guided and installed absorber; tightened bolts, installed rear tail pipe, and reloaded absorber jig. Elevated arm postures: 24%. Most common posture: 135° shoulder flexion. Cycle time: 66.5 s, repetitions: 10, duration: 11 min, 5 s. Team members #3 and #9 (both cassette #3)
15	Team member positioned, aligned, and lifted exhaust components; and tightened connections, sensors, hangers, and heat shield. Elevated arm postures: 45%. Most common posture: 60° shoulder flexion, 90° elbow flexion. Cycle time: 66.5 s, repetitions: 10, duration: 11 min, 5 s. Team member #5 (cassette #3)
16	Team member installed engine components, drive shaft, link arm, front suspension bolts, air spat, and wheel housing plate. Elevated arm postures: 42%. Most common posture: 60° shoulder flexion, 90° elbow flexion. Three stages with cycle time: 66.5 s, repetitions: 3, duration: 9 min, 59 s. Team member #6 (cassette #3)

Each team member was individually fitted with a Levitate Airframe (Levitate Technologies Inc., San Diego, CA). This exoskeleton has a frame, support cassettes, and arm cuffs designed to transfer a portion of the arm and held object weight to the hips. The mechanical support system progressively activates as the arm is raised and gradually releases as the arm is lowered. In its standard configuration, this exoskeleton provides support activation at 30° shoulder flexion, maximum support at 90° shoulder flexion, and continued support until 150° shoulder flexion. The cassettes range in level of support from approximately 10% of the torque created by the arm weight when held horizontally in front of the body (#1 cassette) to 60% of the arm weight torque (#6 cassette). The recommended cassette support is dependent on tool/part weight, tool/part size, frequency of arm lifting/lowering, and size of the team member.

The level of support cassette was chosen so that the arms would lower to a neutral position using gravity rather than applied downward force. This moderate to low level of support was targeted to avoid “fighting” the exoskeleton and fatiguing the shoulder extensor muscles. Team members initially tested the cassette strength by holding their arms at 90° shoulder flexion and letting their arms freely drop. After initial cassette selection, team members were allowed to change cassette strength after working with the exoskeleton during their regular assembly processes. Thirteen team members used a #3 cassette (midrange of increasing cartridge support levels from #1 to #6), while three team members used a #2 cassette. Fittings and adjustments were performed by individuals who had completed a certification program created by the exoskeleton manufacturer.

### 2.2. Experimental protocol

Prior to data collection, each team member provided informed consent as approved by the Institutional Review Board at Iowa State University. Team members practiced using the upper body exoskeleton on the selected processes for at least 1 week before the data collection as part of a larger trial conducted by Toyota. With one exception, data were collected on team members as they performed the assembly processes on 10 consecutive automobiles with and without the exoskeleton. The one exception was a three-stage assembly process that was performed by the team member on four consecutive automobiles with and without the exoskeleton. Depending on the assembly process cycle time, data were collected on each combination of team member, assembly process, and with/without exoskeleton condition for 10–12 min apiece (Table 1). Half of the team members performed the exoskeleton condition first, and half of the team members performed the without exoskeleton condition first. At the conclusion of the data collection, team members completed an exoskeleton usability survey. The survey included an 8.3 cm visual analog scale from −1 (i.e., difficult, uncomfortable, no) to 0 (neutral) to 1 (i.e., easy, comfortable, yes) and prompted users to include qualitative comments.

### 2.3. EMG setup

Data were collected with a Trigno wireless EMG system using EMGworks Acquisition software (Delsys, Natick, MA) at a sampling frequency of 1,926 Hz. EMG sensors were placed bilaterally on the anterior deltoid, biceps brachii, upper trapezius, and lumbar erector spinae muscles following SENIAM recommendations (Hermens et al., 2000). Pre-wrap and athletic tape were used to secure EMG sensor placements. Maximum voluntary isometric contractions (MVICs) were completed in the following postures: 90° shoulder flexion, 90° shoulder abduction, seated elbow flexion, and prone spinal extension. Resistance to movement was applied at the wrist by the researcher during the shoulder flexion, shoulder abduction, and elbow flexion MVICs. During the spinal extension MVIC, resistance to movement was provided by gravity, and the team member’s range of motion limits in a prone position. The MVICs were 5 s in duration, with the first 2 s ramping up to a maximum contraction and the last 3 s at maximum contraction. The team members were given verbal encouragement to generate a maximal voluntary contraction (MVC).

### 2.4. Data analysis

The EMG signals were visually inspected for background noise prior to and during data collection. Prior to processing, any nonphysiological spikes in the data due to occasional contact with the EMG sensor by the exoskeleton or surrounding environment were removed. Data were bandpass filtered from 20 to 450 Hz using a fourth-order zero-lag Butterworth filter, rectified, and low pass filtered at 10 Hz to create a linear envelope. A 1 s moving window was used to find the highest mean EMG amplitude during the MVICs. EMG amplitudes during the assembly processes were averaged over consecutive 1 s intervals and normalized by the MVICs. A muscle was considered active if the EMG amplitude was greater than 5% MVC, and the mean active EMG amplitude was calculated. Duty cycle was determined by dividing the time a muscle was active by the total work time. A muscle’s TLV was then estimated by entering the duty cycle into the equation for upper limb localized fatigue (American Conference of Governmental Industrial Hygienists (ACGIH), 2016). The fatigue risk value was calculated by subtracting the TLV from the mean active EMG amplitude (Gillette and Stephenson, 2019).

Mean active EMG amplitudes and fatigue risk values were determined for the anterior deltoid, biceps brachii, and upper trapezius, with the higher value of the right and left arms retained for analysis. Right and left lumbar erector spinae EMG amplitudes were averaged before determining the mean active EMG amplitudes and fatigue risk values. EMG amplitudes and fatigue risk values were compared for assembly processes performed with the exoskeleton to without the exoskeleton. A positive fatigue risk value indicated that the EMG amplitude was above the TLV for that muscle. Fatigue risk values can be grouped into four categories: (1) reduction with exoskeleton, but still above TLV; (2) reduction with exoskeleton from above to below TLV; (3) reduction with exoskeleton, but process below TLV without exoskeleton; and (4) minimal change or increase with exoskeleton.

The results were collapsed across team members for each assembly process to create a sample size of 16 analyzed. Normality of the dependent variables was assessed using the Kolmogorov–Smirnov statistic. When normally distributed, paired samples *t*-tests were used to compare with exoskeleton to without exoskeleton. When non-normally distributed, Wilcoxon signed ranks tests were used to compare with exoskeleton to without exoskeleton. Statistical significance was set at *p* < .05. If a significant difference in fatigue risk was found for a specific muscle, a secondary analysis was performed to compare posture breakdowns of assembly processes that appeared to benefit more and less from exoskeleton usage. In addition, Cohen’s *d* effect sizes were calculated and interpreted as *d* = 0.20 small effect, *d* = 0.50 medium effect, and *d* = 0.80 large effect (Cohen, 1988). EMG data were analyzed using custom-written Matlab code (MathWorks, Natick, MA), and statistical tests were performed in SPSS version 26 (IBM, Armonk, NY).

## 3. Results

### 3.1. Overall with exoskeleton versus without

Anterior deltoid mean active EMG amplitude, anterior deltoid fatigue risk value, and biceps brachii fatigue risk value were normally distributed and compared using paired samples *t*-tests. The remaining dependent variables were non-normally distributed and compared using Wilcoxen Signed Rank tests.

**Figure 2.**
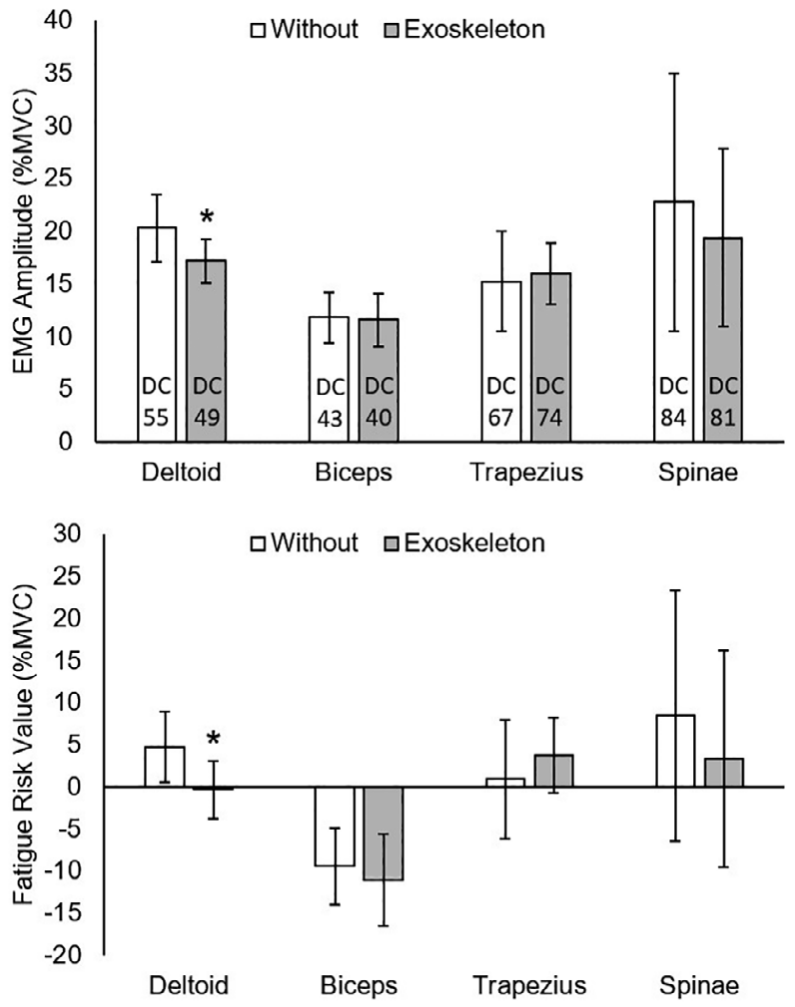
Mean active EMG amplitudes and fatigue risk values without and with the exoskeleton. Vertical bars indicate 95% confidence intervals. DC, duty cycle. *Significant reduction with exoskeleton. Positive fatigue risk values exceed the TLV.

Across the automotive assembly processes, mean active EMG amplitude was significantly reduced with the exoskeleton compared to without for the anterior deltoid (*p* = .01, Δ = −3.2 %MVC, *d* = 0.56 medium effect) (Figure 2). Fatigue risk value was also significantly reduced with the exoskeleton compared to without for the anterior deltoid (*p* < .01, Δ = −5.1 %MVC, *d* = 0.62 medium effect) across the assembly processes. Mean active EMG amplitudes were not significantly changed with the exoskeleton compared to without for the biceps brachii (*p* = .80, Δ = −0.2 %MVC, *d* = 0.05), upper trapezius (*p* = .28, Δ = 0.8 %MVC, *d* = 0.10), or lumbar erector spinae (*p* = .30, Δ = −3.4 %MVC, *d* = 0.16). Similarly, fatigue risk values were not significantly changed with the exoskeleton compared to without for the biceps brachii (*p* = .47, Δ = −1.7 %MVC, *d* = 0.17), upper trapezius (*p* = .09, Δ = 2.8 %MVC, *d* = 0.24 small effect), or lumbar erector spinae (*p* = .15, Δ = −5.1 %MVC, *d* = 0.18).

### 3.2. Grouped assembly processes with exoskeleton versus without

Since there was a significant reduction in anterior deltoid fatigue risk value, these results were further examined to determine if certain automotive assembly processes appear to be a better fit for exoskeleton usage than others. A reduction in fatigue risk value of at least 2 %MVC was considered meaningful since it would ensure at least a 10% reduction in fatigue risk relative to the TLV at a duty cycle of 50%. Using this guideline, (1) four assembly processes had a reduced fatigue risk value with the exoskeleton, but were still above the TLV (processes #1–#4); (2) five processes had a reduced fatigue risk with the exoskeleton from above to below the TLV (processes #5–#9); (3) four processes had a reduced fatigue risk with the exoskeleton, but the process was below the TLV without the exoskeleton (processes #13–#16); and (4) three processes had a minimal reduction or increase in fatigue risk with the exoskeleton (processes #10–#12). The four assembly processes from category one were combined with the five processes from category two to create a comparison group that appears to benefit the most from exoskeleton usage. Likewise, the four processes from category three were combined with the three processes from category four to create a group that appears to benefit less from exoskeleton usage.

**Figure 3.**
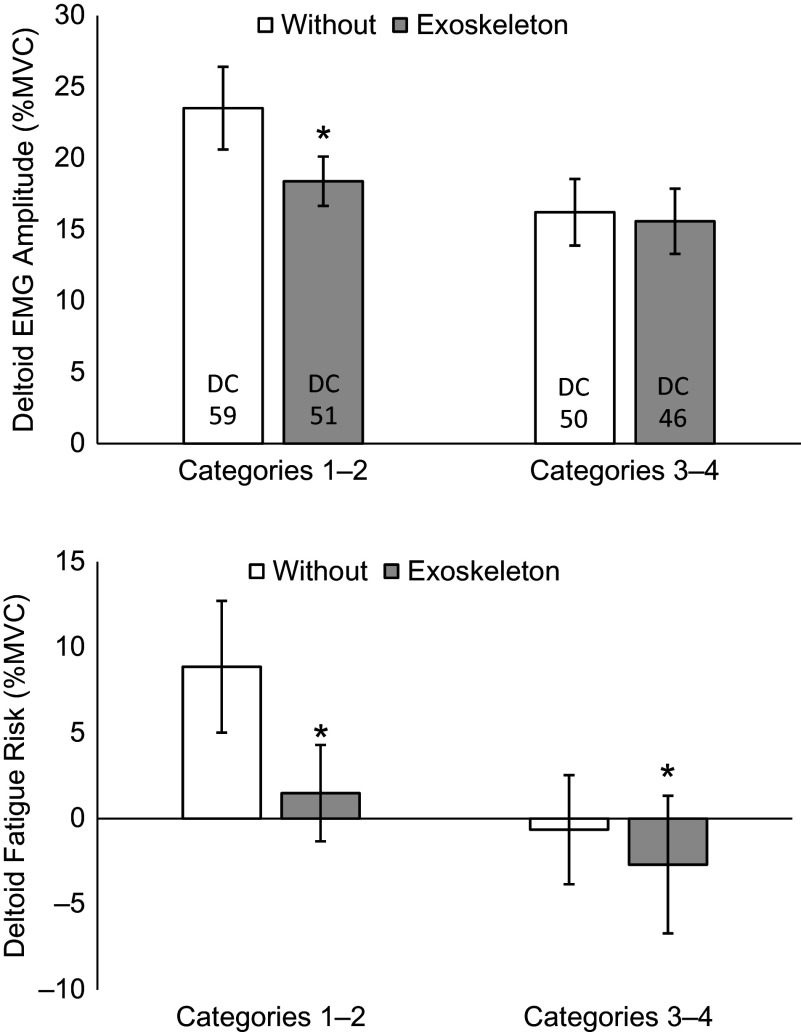
Mean active EMG amplitudes and fatigue risk values for the anterior deltoid without and with the exoskeleton. Vertical bars indicate 95% confidence intervals. DC, duty cycle. *Significant reduction with exoskeleton. Positive fatigue risk values exceed the TLV.

Anterior deltoid mean active EMG amplitudes and fatigue risk values were normally distributed for combined categories one and two and for combined categories three and four, and thus were compared using paired samples *t*-tests. For categories one and two, mean active EMG amplitude for the anterior deltoid was significantly reduced with the exoskeleton compared to without (*p* < .01, Δ = −5.1 %MVC, *d* = 0.95 large effect) (Figure 3). Fatigue risk value for the anterior deltoid was significantly reduced with the exoskeleton for both categories one and two (*p* < .01, Δ = −7.4 %MVC, *d* = 0.96 large effect) and categories three and four (*p* = .04, Δ = −2.0 %MVC, *d* = 0.28 small effect). Mean active EMG amplitude for the anterior deltoid was not significantly changed with the exoskeleton for categories three and four (*p* = .38, Δ = −1.2 %MVC, *d* = 0.14).

The posture breakdowns for each process provided by Toyota were analyzed and averaged for categories one and two and categories three and four. Shoulder elevation angles were grouped into percent time at or above 60°, percent time at or above 90°, and percent time at or above 135°. Team members spent a similar time ≥60° shoulder elevation for categories one and two (37%) and categories three and four (38%). However, team members spent a greater amount of time ≥90° shoulder elevation for categories one and two (30%) compared to categories three and four (24%). This difference was largest at the highest shoulder elevations, where team members spent a greater amount of time ≥135° shoulder elevation for categories one and two (18%) than for categories three and four (9%).

### 3.3. Exoskeleton usability survey

For the exoskeleton usability survey, positive scores were considered favorable responses, while negative scores were considered unfavorable responses (Table 2). The highest survey values were 0.78 for “Would you suggest others use the exoskeleton?” (−1 no, 1 yes), 0.53 for “How physically comfortable were you with the exoskeleton?” (−1 uncomfortable, 1 comfortable), 0.52 for “After adjustment, how comfortable did you find the exoskeleton?’ (−1 uncomfortable, 1 comfortable), and 0.50 for ‘Would you choose to use the exoskeleton for your job duties again?” (−1 no, 1 yes). One area of concern was a −0.04 for “Did the exoskeleton hinder you in doing your job duties in any way?” (−1 yes, 1 no).

**Table 2.** Exoskeleton usability survey results

Question (scale)	Mean (SD)
How easy did you find the exoskeleton to put on and adjust? (−1 = Difficult, 0 = Neutral, 1 = Easy)	0.34 (0.65)
After adjustment, how comfortable did you find the exoskeleton? (−1 = Uncomfortable, 0 = Neutral, 1 = Comfortable)	0.52 (0.32)
How difficult was your job without the exoskeleton? (−1 = Difficult, 0 = Neutral, 1 = Easy)	−0.08 (0.54)
How difficult was your job with the exoskeleton? (−1 = Difficult, 0 = Neutral, 1 = Easy)	0.46 (0.56)
How physically comfortable were you without the exoskeleton? (−1 = Uncomfortable, 0 = Neutral, 1 = Comfortable)	0.41 (0.56)
How physically comfortable were you with the exoskeleton? (−1 = Uncomfortable, 0 = Neutral, 1 = Comfortable)	0.53 (0.33)
Did the exoskeleton hinder you in doing your job duties in any way? (−1 = Yes, 0 = Neutral, 1 = No)	−0.04 (0.77)
Overall, was the exoskeleton a benefit to your job duties? (−1 = No, 0 = Neutral, 1 = Yes)	0.47 (0.56)
Would you choose to use the exoskeleton for your job duties again? (−1 = No, 0 = Neutral, 1 = Yes)	0.50 (0.68)
Would you suggest others use the exoskeleton? (−1 = No, 0 = Neutral, 1 = Yes)	0.78 (0.45)

## 4. Discussion

The main purpose of this study was to assess an upper body exoskeleton during automotive assembly processes that involve elevated arm postures. A secondary goal was to examine which assembly processes may benefit more from exoskeleton usage. As will be discussed, the upper body exoskeleton reduced anterior deltoid fatigue risk overall, while specific assembly processes appear to benefit more than others.

Our hypothesis that anterior deltoid activation and fatigue risk would be significantly reduced with the exoskeleton was supported. Reduced anterior deltoid activation is consistent with the design of the exoskeleton supporting shoulder flexion. In addition, the ergonomic assessments provided by Toyota indicated that the assembly processes tested required shoulder angles of 60° or greater for 24% up to 73% of the duty cycle (Table 1). Reduced anterior deltoid activation is consistent with previous studies that assessed exoskeletons during lab-based simulations or on-site job tasks (Kim et al., 2018a; Alabdulkarim et al., 2019; Alabdulkarim and Nussbaum, 2019; Kim and Nussbaum, 2019; Van Engelhoven et al., 2019; Iranzo et al., 2020). Reduction in fatigue risk can be attributed to both a reduction in EMG amplitude and a reduction in duty cycle (Figure 2), which increased the fatigue TLV (American Conference of Governmental Industrial Hygienists (ACGIH), 2016). This finding is consistent with a previous on-site study (Gillette and Stephenson, 2019). Thus, the anterior deltoid had both lower activation levels and was active less often for a combined benefit when using the exoskeleton.

The biceps brachii, trapezius descendens, and erector spinae activation and fatigue risk were not significantly changed. Thus, reduced anterior deltoid activity was the primary benefit of exoskeleton usage for the assembly processes tested. Surrounding muscles did not increase activity to offset the anterior deltoid reduction, but also did not directly benefit from exoskeleton usage. Similar exoskeletons have been found to either reduce (Kim et al., 2018a; Schmalz et al., 2019; Iranzo et al., 2020) or increase trapezius activity (de Vries et al., 2019). An explanation may be that moderate arm elevation angles (60°–135°) benefit from exoskeleton arm support, but higher angles create resistance to scapular elevation. Exoskeletons have also been found to reduce lumbar erector spinae and latissimus dorsi muscle activity (Butler and Gillette, 2019; de Vries et al., 2019). An exoskeleton may provide benefits if it reduces trunk lean when a tool/part is held in front of the body or may increase low back activity if the exoskeleton is heavy.

Four automotive assembly processes were considered category one and had a reduced anterior deltoid fatigue risk value with the exoskeleton, but were still above the TLV. These processes appear to benefit from exoskeleton usage, but may also require additional engineering controls to prevent fatigue. It is possible that increasing the exoskeleton cassette level would be a solution, but antagonist muscles would need to be tested for fatigue with increased resistance lowering the arms. Five processes were considered category two and had a reduced anterior deltoid fatigue risk value with the exoskeleton from above to below the TLV. These processes appear to be the most promising fit as the risk prediction goes from fatiguing to non-fatiguing with exoskeleton usage. These nine processes were grouped together as job tasks that appear to benefit the most from exoskeleton usage.

Four assembly processes were considered category three and had reduced anterior deltoid fatigue risk with the exoskeleton, but the process was below the TLV without the exoskeleton. These processes may benefit from exoskeleton usage, but are not predicted to cause fatigue in the anterior deltoid, so would likely be lower priority. The decision on further exoskeleton testing for adoption may be based on risk aversion and if an acceptable safety margin exists below the TLV. Finally, three processes were considered category four and had minimal change (>2 %MVC) in anterior deltoid fatigue risk with the exoskeleton. These processes may require adjustments to range of support or support level, a different design/type of exoskeleton, or may not be a good fit for exoskeleton usage. These seven processes were grouped together as job tasks that appear benefit less or to not benefit from exoskeleton usage.

The processes in categories one and two had a significant reduction in anterior deltoid activation, while the processes in categories three and four did not (Figure 3). Both groups had a significant reduction in anterior deltoid fatigue risk, although the processes in categories one and two had a much larger reduction. When comparing posture breakdowns, both process groups involved similar percentage of time in shoulder elevations at or above 60°. In contrast, processes in categories one and two require a greater percentage time in shoulder elevations at or above 90° and at or above 135°. These results indicate the importance of considering durations in arm postures, with this exoskeleton appearing to be most effective in the 90°–135° range of shoulder elevation. Additional factors such as tool/part weight and applied force likely further determine whether a process falls within category one versus two or category three versus four.

Team members were positive about recommending the exoskeleton to others. Qualitative comments indicated that they thought the exoskeleton would reduce strain, increase energy, and would be particularly beneficial for processes with extensive overhead work and for new employees. Survey results were positive overall about the comfort and adjustability of the exoskeleton. Team members stressed the importance of individually fitting the exoskeleton. One survey area where the exoskeleton was judged as neutral was hindering job duties. Some team members expressed concern that the exoskeleton could make it difficult to pick up dropped objects, could get caught when working in tight spaces, and could restrict twisting movements. Across categories, team members suggested that the exoskeleton had potential to reduce fatigue if fitted properly and used with overhead work in relatively open spaces.

There are limitations to this study. First, while 16 team members participated in this study, the majority of assembly processes were performed by only two participants. Further studies with higher participant numbers for each process may allow exoskeleton recommendations for individual processes and would improve generalizability. Second, interpretation of the results applies to processes that involve combinations of postures similar to those tested. Further lab-based studies could help determine optimal ranges of postures and tool weights for exoskeleton usage. Third, the results reflect processes performed with the Levitate Airframe in the standard configuration with predominantly #3 cassettes. Other upper body exoskeleton designs with higher/lower support over different ranges of motion may result in different effects on fatigue risk and worker opinions of usability. Fourth, the accuracy of the ACGIH TLV method was dependent upon the team member’s level of effort during the maximal contractions and a low level of background noise in the EMG signals. Job tasks with higher levels of background noise may require adjustment of filtering parameters (i.e., welding, Gillette and Stephenson, 2019) or different processing algorithms (i.e., Micera et al., 2001). Fifth, the ACGIH TLV curves were designed for upper limb generalized fatigue, so this method should be used with caution for back muscles. Additional use of a structural fatigue model is recommended if estimated low back compressive forces are of concern for a job task (i.e., Zelik et al., 2022). Finally, this study tested acute fatigue, and further long-term studies of exoskeleton comfort and injury prevention are recommended.

## 5. Conclusion

The exoskeleton reduced anterior deltoid muscle activity and fatigue risk overall, without increasing the burden on other muscles tested. Assembly processes that involved a greater amount of time in arm elevations above 90° appeared to benefit the most from exoskeleton usage. Team members responded positively overall about exoskeleton usage, especially for comfort and potential benefit to workers. However, there were some concerns about the exoskeleton potentially hindering certain job duties. The results support the importance of testing different assembly processes to match the mechanical benefits of an exoskeleton. In addition, the survey results stress the importance of fitting and trialing an exoskeleton with job tasks.

## Data Availability

The data that support the findings of this study are available from the corresponding author, J.C.G. upon reasonable request.
